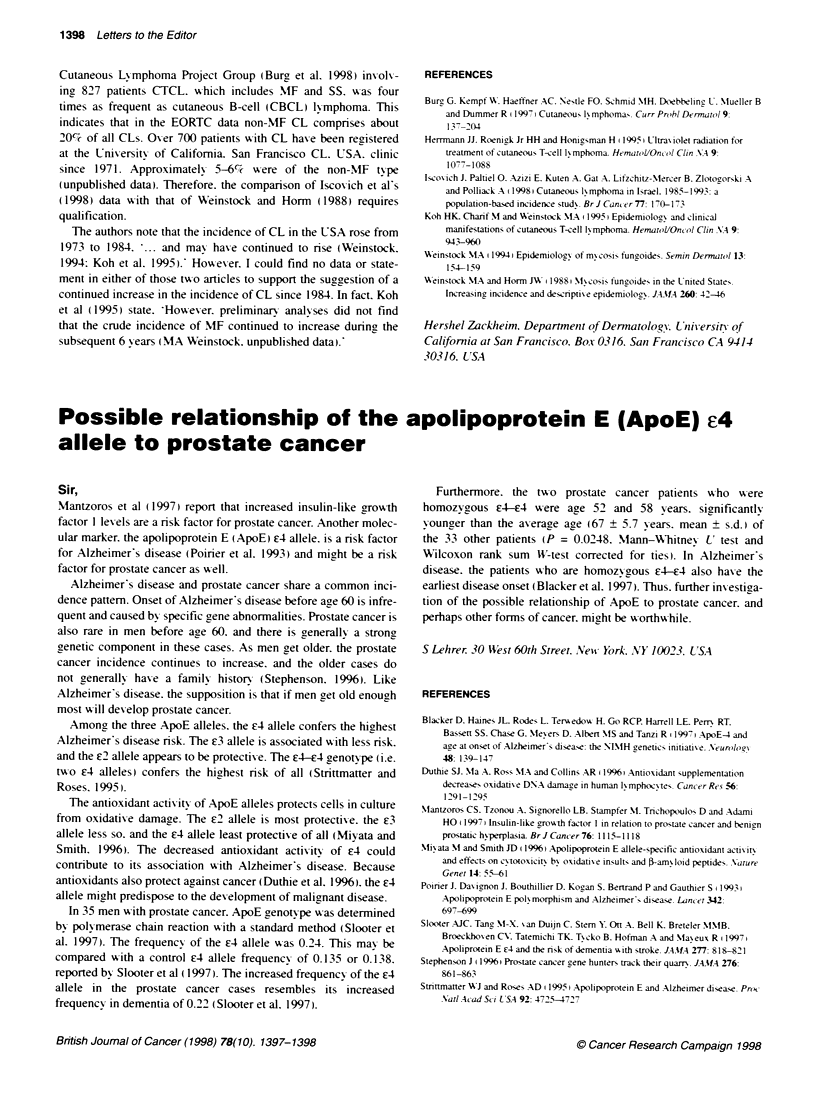# Possible relationship of the apolipoprotein E (ApoE) epsilon4 allele to prostate cancer.

**DOI:** 10.1038/bjc.1998.695

**Published:** 1998-11

**Authors:** S. Lehrer


### Possible relationship of the apolipoprotein E (ApoE) ε4 allele to prostate cancer

**Sir,**

Mantzoros et al (1997) report that increased insulin-like growth factor 1 levels are a risk factor for prostate cancer. Another molecular marker, the apolipoprotein E (ApoE) ε4 allele, is a risk factor for Alzheimer's disease (Poirier et al, 1993) and might be a risk factor for prostate cancer as well.

Alzheimer's disease and prostate cancer share a common incidence pattern. Onset of Alzheimer's disease before age 60 is infrequent and caused by specific gene abnormalities. Prostate cancer is also rare in men before age 60, and there is generally a strong genetic component in these cases. As men get older, the prostate cancer incidence continues to increase, and the older cases do not generally have a family history (Stephenson, 1996). Like Alzheimer's disease, the supposition is that if men get old enough most will develop prostate cancer.

Among the three ApoE alleles, the ε4 allele confers the highest Alzheimer's disease risk. The ε3 allele is associated with less risk, and the ε2 allele appears to be protective. The ε4–ε4 genotype (i.e. two ε4 alleles) confers the highest risk of all (Strittmatter and Roses, 1995).

The antioxidant activity of ApoE alleles protects cells in culture from oxidative damage. The ε2 allele is most protective, the ε3 allele less so, and the ε4 allele least protective of all (Miyata and Smith, 1996). The decreased antioxidant activity of ε4 could contribute to its association with Alzheimer's disease. Because antioxidants also protect against cancer (Duthie et al, 1996), the ε4 allele might predispose to the development of malignant disease.

In 35 men with prostate cancer, ApoE genotype was determined by polymerase chain reaction with a standard method (Slooter et al, 1997). The frequency of the ε4 allele was 0.24. This may be compared with a control ε4 allele frequency of 0.135 or 0.138, reported by Slooter et al (1997). The increased frequency of the ε4 allele in the prostate cancer cases resembles its increased frequency in dementia of 0.22 (Slooter et al, 1997).

Furthermore, the two prostate cancer patients who were homozygous ε4–ε4 were age 52 and 58 years, significantly younger than the average age (67 ± 5.7 years, mean ± s.d.) of the 33 other patients ($P$ = 0.0248, Mann–Whitney $U$ test and Wilcoxon rank sum $W$-test corrected for ties). In Alzheimer's disease, the patients who are homozygous ε4–ε4 also have the earliest disease onset (Blacker et al, 1997). Thus, further investigation of the possible relationship of ApoE to prostate cancer, and perhaps other forms of cancer, might be worthwhile.

*S Lehrer, 30 West 60th Street, New York, NY 10023, USA*

**REFERENCES**

Blacker D, Haines JL, Rodes L, Terwedow H, Go RCP, Harrell LE, Perry RT, Bassett SS, Chase G, Meyers D, Albert MS and Tanzi R (1997) ApoE-4 and age at onset of Alzheimer's disease: the NIMH genetics initiative. *Neurology* **48**: 139–147

Duthie SJ, Ma A, Ross MA and Collins AR (1996) Antioxidant supplementation decreases oxidative DNA damage in human lymphocytes. *Cancer Res* **56**: 1291–1295

Mantzoros CS, Tzonou A, Signorello LB, Stampfer M, Trichopoulos D and Adami HO (1997) Insulin-like growth factor 1 in relation to prostate cancer and benign prostatic hyperplasia. *Br J Cancer* **76**: 1115–1118

Miyata M and Smith JD (1996) Apolipoprotein E allele-specific antioxidant activity and effects on cytotoxicity by oxidative insults and β-amyloid peptides. *Nature Genet* **14**: 55–61

Poirier J, Davignon J, Bouthillier D, Kogan S, Bertrand P and Gauthier S (1993) Apolipoprotein E polymorphism and Alzheimer's disease. *Lancet* **342**: 697–699

Slooter AJC, Tang M-X, van Duijn C, Stern Y, Ott A, Bell K, Breteler MMB, Broeckhoven CV, Tatemichi TK, Tycko B, Hofman A and Mayeux R (1997) Apoliprotein E ε4 and the risk of dementia with stroke. *JAMA* **277**: 818–821

Stephenson J (1996) Prostate cancer gene hunters track their quarry. *JAMA* **276**: 861–863

Strittmatter WJ and Roses AD (1995) Apolipoprotein E and Alzheimer disease. *Proc Natl Acad Sci USA* **92**: 4725–4727

© Cancer Research Campaign 1998